# Transthyretin Amyloid Cardiomyopathy—2025 Update: Current Diagnostic Approaches and Emerging Therapeutic Options

**DOI:** 10.3390/jcm14134785

**Published:** 2025-07-07

**Authors:** Carsten Tschöpe, Ahmed Elsanhoury, Arnt V. Kristen

**Affiliations:** 1 Department of Cardiology, Angiology, and Intensive Medicine (CVK), German Heart Center at Charité (DHZC), Charité—University Medicine Berlin, 13353 Berlin, Germany; 2 Berlin Institute of Health (BIH), Center for Regenerative Therapies (BCRT), Charité—University Medicine Berlin, 13353 Berlin, Germany; ahmed.elsanhoury@bih-charite.de; 3 Amyloidosis Center, Charité—Universitätsmedizin Berlin (ACCB), Corporate Member of Freie Universität Berlin and Humboldt-Universität zu Berlin, Charitéplatz 1, 10117 Berlin, Germany; 4 German Centre for Cardiovascular Research (DZHK), Partner Site Berlin, 13353 Berlin, Germany; 5 Department of Cardiology, Angiology and Respiratory Medicine, University Hospital Heidelberg, 69120 Heidelberg, Germany; kristen@kardio-darmstadt.de; 6 Kardiovaskuläres Zentrum Darmstadt, 64287 Darmstadt, Germany

**Keywords:** amyloidosis, transthyretin, cardiac amyloidosis, ATTR cardiomyopathy, ATTR polyneuropathy, clinical development, antisense oligonucleotide, siRNA, gene editing

## Abstract

Transthyretin-related (ATTR) amyloidosis is a progressive, multisystem disease caused by the extracellular deposition of misfolded transthyretin (TTR) monomers as insoluble amyloid fibrils. Clinical manifestations vary widely and may include cardiomyopathy (ATTR-CM), polyneuropathy (ATTR-PN), or mixed phenotypes. The condition is increasingly recognized as an underdiagnosed contributor to heart failure, particularly in elderly patients. ATTR amyloidosis exists in two major forms: hereditary (ATTRv), resulting from mutations in the TTR gene, and wild-type (ATTRwt), typically affecting men over 70 years of age. Advances in disease understanding have led to a paradigm shift in management, with the introduction of targeted therapies that slow disease progression and improve prognosis. First-generation therapies such as tafamidis have demonstrated survival benefits in ATTR-CM. More recently, second-generation agents—such as the TTR stabilizer acoramidis and RNA silencers including vutrisiran and eplontersen—have shown promising efficacy in clinical trials. Additional strategies under investigation include gene editing and monoclonal antibodies targeting TTR amyloid deposits. This review outlines current diagnostic strategies and therapeutic options for ATTR amyloidosis, emphasizing the need for early detection and individualized treatment approaches. The expanding therapeutic landscape highlights the importance of accurate phenotyping and timely intervention to optimize clinical outcomes.

## 1. Introduction

Cardiac amyloidosis remains underdiagnosed despite its increasing recognition. The prevalence, particularly of transthyretin-related forms, rises with age and comorbidities such as heart failure with preserved ejection fraction (HFpEF). Epidemiological estimates suggest a prevalence of up to 13% among older HFpEF (EF ca. 50%) patients with severe left ventricular hypertrophy (LV). Although HFpEF is the predominant phenotype in ATTR amyloidosis, up to one third of patients may exhibit a transition to heart failure with reduced EF (HFrEF, LVEF < 30%) in advanced stages. Cardiac involvement is associated with poor prognosis, especially when accompanied by renal impairment. This review aims to summarize current concepts in the classification, diagnosis, and treatment of cardiac amyloidosis with a focus on practical clinical implementation.

Amyloidosis is a heterogeneous group of diseases caused by the extracellular deposition of misfolded amyloid protein aggregates, leading to progressive tissue damage and organ dysfunction [1,2,3]. Over 40 different amyloid fibril proteins have been identified as causing amyloidosis in humans, of which 19 are associated with systemic deposition. The most common types of systemic amyloidosis include immunoglobulin light chain (AL) amyloidosis, serum amyloid A (AA) amyloidosis, and transthyretin (ATTR) amyloidosis [2]. Amyloidosis subtypes differ by precursor protein, organ tropism, and therapeutic options. Wild-type and hereditary ATTR mainly affect the heart, whereas AL also involves the kidneys, and AA is systemic and inflammation-driven.

ATTR amyloidosis exists in two distinct forms: hereditary and wild-type ATTR amyloidosis. The hereditary form (ATTRv; v for “variant”) is a progressive, autosomal dominant disorder caused by mutations in the transthyretin (TTR) gene [4]. There are more than 130 known variants, with considerable differences in regional prevalence and the resulting phenotype depending on the site and extent of deposition [4,5,6]. The phenotype spectrum ranges from ATTR variants almost exclusively associated with cardiomyopathy (ATTR-CM) to those with predominant polyneuropathy (ATTR-PN) [4,7,8]. In wild-type ATTR amyloidosis (ATTRwt), an age-related condition previously known as “senile systemic amyloidosis”, the amyloid fibrils consist of normal, non-mutated TTR proteins [8,9,10]. Patients with the ATTRwt phenotype are typically males (>90%) of older age (>70 years) presenting with predominant ATTR-CM [5,8,11]. Genetic testing is crucial to distinguish between ATTRv and ATTRwt amyloidosis [4,12].

**Figure 1:**
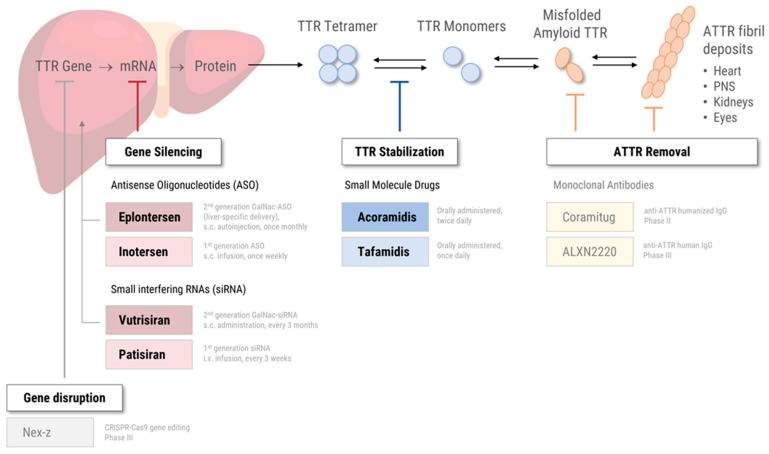
Pathogenesis and specific therapeutic strategies in transthyretin (ATTR) amyloidosis. The figure illustrates the production of TTR protein in the liver, its dissociation from tetramers into monomers, misfolding, and aggregation into amyloid fibrils that deposit in the heart, peripheral nervous system (PNS), kidneys, and eyes. Three therapeutic strategies are shown: (1) Gene silencing, using antisense oligonucleotides (ASOs) and small interfering RNAs (siRNAs); (2) TTR stabilization, with small-molecule drugs that prevent tetramer dissociation; and (3) ATTR removal, with monoclonal antibodies targeting misfolded TTR or amyloid deposits. → indicates biological progression; ⇌ indicates reversible molecular equilibrium; ⊥ indicates pharmacological inhibition at the respective stage. Abbreviations: ASO, antisense oligonucleotide; CRISPR, clustered regularly interspaced short palindromic repeats; GalNAc, N-acetylgalactosamine; IgG, immunoglobulin G; i.v., intravenous; PNS, peripheral nervous system; s.c., subcutaneous; siRNA, small interfering RNA.

Given the central role of transthyretin in ATTR amyloidosis, understanding its structure and function is essential. Transthyretin (TTR) is a 55 kDa protein mainly produced by hepatocytes in the liver and to a lesser extent by the choroid plexus and retinal epithelium [4,13]. The main physiological function of TTR is the transport of thyroxine (T4) and retinol bound to retinol-binding protein (RBP) [13]. TTR has a tetrameric structure composed of four identical β-sheet-rich subunits with two T4 and four RBP-binding sites [13,14]. In ATTR amyloidosis, the TTR tetramers are destabilized, either due to structural changes resulting from genetic mutations (in ATTRv) or yet unknown age-related factors (in ATTRwt amyloidosis) [4,14]. The resulting TTR monomers tend to misfold and aggregate into insoluble ATTR amyloid fibrils [3,15,16]. These fibril deposits accumulate in the extracellular space of various tissues and organs, most commonly the heart, the peripheral nervous system, the kidneys, and the eyes (Figure 1) [12,17,18,19].

ATTR amyloidosis is a multisystemic and progressive disease that is fatal if left untreated, with a median survival of 8 to 10 years for ATTRv and 3 to 4 years for ATTR-CM after disease onset [20]. Diagnosis can be challenging as patients present with a heterogeneous and nonspecific spectrum of symptoms [12,18,19]. Patients with cardiac manifestations typically experience progressive HFpEF due to amyloid deposition in the myocardium, resulting in restrictive hypertrophic cardiomyopathy, among other cardiac conditions (Section 2.1). Patients with significant deposition in the peripheral and autonomic nervous systems present with sensory-motor impairments and autonomic dysfunction (Section 2.2). Historically, symptomatic patients are classified by their primary symptoms and predominant phenotype into ATTR-CM or ATTR-PN. However, around 33% of symptomatic patients worldwide have a mixed phenotype exhibiting both cardiac and neurological manifestations, further complicating accurate diagnosis and management (Section 2.3). Furthermore, ATTR amyloidosis is subclassified by genotype, as different TTR gene mutations lead to varying clinical manifestations, disease severity, and organ involvement [5,21]. Although considered a rare disease, with an estimated global prevalence of 200,000–300,000 persons for ATTRwt and 10,000–40,000 for ATTRv, recent data suggest that the prevalence of ATTR amyloidosis may be as high as 15% in certain subgroups, e.g., elderly patients with aortic stenosis, LVH, or HFpEF [9,22,23,24]. A timely diagnosis is essential, due to the progressive nature and the emergence of disease-modifying therapies, which can slow progression [19].

This review provides an overview of current diagnostic approaches (Section 2), specific therapies approved for ATTRv and ATTRwt amyloidosis (Section 3 and Section 4), and future therapeutic options (Section 5), with a focus on patients with ATTR-CM, including mixed phenotype patients with additional ATTR-PN. We also highlight the need for ongoing research to address unmet medical needs (Section 6), including the improvement of diagnostic tools for earlier detection, the optimization of existing treatment strategies to better accommodate the heterogeneous nature of the disease and enable personalized treatment approaches, and the development of new therapeutic options. Specific therapies should effectively slow disease progression, improve survival, and enhance the health-related quality of life, while minimizing side effects and barriers to adherence.

## 2. Clinical Manifestations and Diagnosis

The Transthyretin Amyloidosis Outcomes Survey (THAOS; NCT00628745) is a global, longitudinal observational registry study including patients with ATTR amyloidosis and asymptomatic TTR mutation carriers between 2007 and 2023 [5]. A recent analysis of 4428 symptomatic patients showed that in the case of ATTRv, the p.Val50Met variant is by far the most common genotype worldwide. 48% of the patients were p.Val50Met positive, followed by p.Val142lle (6%) and p.Glu109Gln (2.4%). ATTRwt was present in 25% of the cases. However, there are significant regional differences, e.g., while p.Val50Met is the most common genotype in Asia (48%), Europe (54%), and South America (79%); patients with ATTRwt amyloidosis were most commonly enrolled in North America (59%) [5]. In line with epidemiological studies worldwide, most patients are male (71%) across all genotypes, with a mean age of 57 years at symptom onset [5,11]. ATTRwt amyloidosis patients are almost exclusively male (>90%) with later disease onset (>70 years) [5,11]. Approximately 32% of all symptomatic patients are classified as predominant ATTR-CM and 39% as predominant ATTR-PN, whereas 24.5% belong to the mixed phenotype category [5]. Although ATTRwt patients typically present with cardiac amyloidosis, a significant proportion in Europe (30%) and North America (50%) have additional polyneuropathic symptoms [5]. Cardiologists must therefore be aware of neurological red flags that should raise additional suspicion of ATTR amyloidosis [19,25,26,27]. For additional considerations regarding the diagnosis of mixed phenotype patients, see Section 2.3.

### 2.1. ATTR Cardiomyopathy (ATTR-CM)

In most patients with ATTRwt, as well as in selected ATTRv cases (e.g., p.Val142Ile, p.Thr80Ala, and p.Ile88Leu), amyloid fibrils predominantly accumulate in the myocardium [5,6]. Notably, all symptomatic ATTRwt patients in the THAOS registry are classified as either ATTR-CM (76%) or mixed phenotype (24%) [5]. Cardiac amyloidosis leads to restrictive cardiomyopathy with a concentric LV hypertrophy (≥12 mm). Involvement of the cardiac valves and conduction system contributes to arrhythmias and heart failure (HF) [8,10]. LV is most commonly affected; however, the right ventricle (RV) and atrial walls can also be involved [8,10,19]. Since patient outcome highly depends on the timely initiation of disease-modifying therapy, the diagnosis should be confirmed as soon as cardiac amyloidosis is suspected [19].

#### 2.1.1. Typical Symptoms and Cardiac Red Flags

Patients with cardiac amyloidosis typically present with a group of cardiac manifestations (Table 1) that should raise suspicion of ATTR-CM [19,25,26,27,28,29]. Extracardiac red flags mainly result from amyloid depositions in the musculoskeletal tissue and the peripheral and autonomic nervous system. The typical polyneuropathic symptoms, orthopedic manifestations, and autonomic dysfunctions of ATTR-PN and mixed phenotype patients are described in Section 2.3 (Table 2).

Cardiac red flags include clinical symptoms of HF, aortic stenosis, persistently increased cardiac troponin level, and unusually high NT-proBNP to the degree of heart failure. Cardiopulmonary exercise testing can complement functional assessment in cardiac amyloidosis, especially for evaluating therapy response or differential diagnosis in early disease stages. Typical findings in echocardiogram (ECHO) include a granular sparkling pattern of the myocardium, LV wall thickening (sometimes with coexisting RV and valve thickness), longitudinal dysfunction with preserved strain in the left ventricular apex (known as “apical sparing”), and pericardial effusion. In echocardiography, left atrial strain analysis has emerged as a useful parameter in distinguishing cardiac amyloidosis from other hypertrophic phenotypes, particularly when apical sparing is absent. Novel echocardiographic tools, such as myocardial work analysis, can provide additional information about myocardial efficiency and strain patterns in suspected cardiac amyloidosis and may help distinguish it from hypertrophic cardiomyopathy or hypertensive heart disease. Emerging echocardiographic techniques, such as myocardial work indices, may offer additional value in characterizing myocardial mechanics and treatment response. Abnormal electrocardiogram (ECG) findings comprise pseudo-infarction patterns with prolonged QTc interval, low QRS voltage (disproportionately low to LV thickening), and atrioventricular (AV) conduction abnormalities. Characteristic features in cardiac magnetic resonance (CMR) imaging are abnormal gadolinium kinetics and typical late gadolinium enhancement (LGE) patterns, e.g., subendocardial or transmural LGE, and highly increased native T1 (Table 1) [19,25,26,27,28,29]. In addition, extracellular volume (ECV) quantification derived from T1 mapping allows estimation of interstitial expansion and can reflect amyloid infiltration. Elevated ECV values are typical in ATTR-CM and can help differentiate it from other causes of left ventricular hypertrophy.

#### 2.1.2. Differential Diagnosis

ATTR-CM is frequently misdiagnosed with other cardiac diseases. Therefore, ATTR-CM should be differentiated from hypertrophic cardiomyopathy (HCM), other restrictive cardiomyopathies, or other cardiac amyloidosis (e.g., AL or AA amyloidosis) and storage disorders (e.g., Fabry, Danon, Pompe or Gaucher disease) [19,20,27,30]. However, >98% of patients with cardiac amyloidosis are diagnosed with either AL or ATTR amyloidosis [27]. Of note, according to a recent meta-analysis, ATTR-CM was diagnosed in 7% of patients with LV wall thickness ≥15 mm, 12% of HFpEF patients, and 10–15% of elderly patients with AS undergoing valve replacement [31].

#### 2.1.3. Diagnostic Algorithm

**Figure 2:**
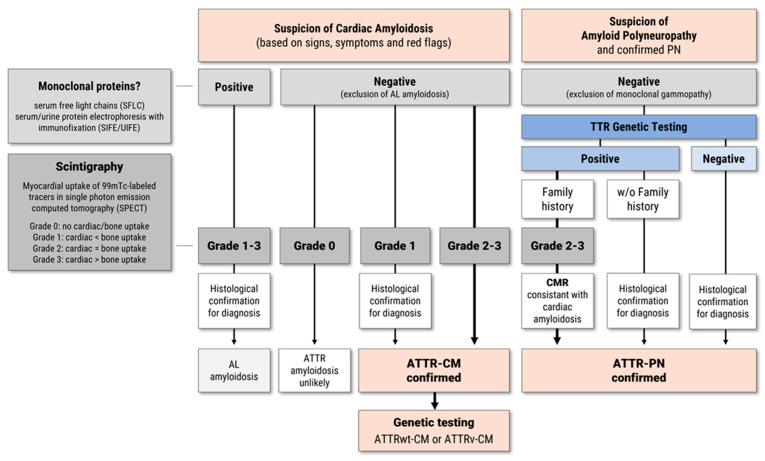
Possible diagnostic algorithm for ATTR-CM and ATTR-PN [17,18,19,30]. The figure illustrates a stepwise diagnostic pathway for suspected transthyretin amyloidosis (ATTR) presenting with either cardiac (ATTR-CM) and/or neurological (ATTR-PN) symptoms. Evaluation includes laboratory screening for monoclonal proteins, bone scintigraphy (graded 0–3), histological confirmation, and genetic testing for hereditary ATTR variants. Cardiac magnetic resonance (CMR) may support the diagnosis in patients with polyneuropathy and suggest cardiac involvement. Abbreviations: AL, light-chain (amyloid) amyloidosis; ATTR, transthyretin amyloidosis; CM, cardiomyopathy; PN, polyneuropathy.

Usually, cardiac amyloidosis is confirmed by histological staining after endomyocardial biopsy (EMB) and subsequent identification of the amyloid protein using immunohistochemistry (IHC) or mass spectrometry (MS) [27,30]. Despite advances in imaging, Congo red staining of biopsy specimens remains the diagnostic gold standard, particularly for AL amyloidosis. In clinical practice, a non-invasive diagnostic pathway based on typical cardiac imaging findings and positive bone scintigraphy, in the absence of monoclonal gammopathy, can suffice for the diagnosis of ATTR-CM. In patients with typical signs of ATTR-CM on ECHO and CMR imaging, the diagnosis of ATTR-CM is considered confirmed if the following criteria are met: scintigraphy using 99mTc-labeled tracers shows Grade 2 or Grade 3 myocardial uptake in single photon emission computed tomography (SPECT) equal to or greater than bone uptake and if AL amyloidosis can be excluded by negative testing for monoclonal proteins using serum free light chain (SFLC) assay, and serum and urine protein electrophoresis with immunofixation (SIFE/UIFE) (Figure 2) [19,20,27,30]. For patients with normal monoclonal protein levels and Grade 1 cardiac uptake, ATTR-CM diagnosis should be confirmed by cardiac or extracardiac biopsy and IHC/MS [19,20,27,30]. Subsequent genetic testing for TTR gene mutations is mandatory for all patients with confirmed ATTR-CM diagnosis to differentiate between ATTRv and ATTRwt (Figure 2). For patients with confirmed ATTRv mutation, genetic counseling and family screening are recommended for all first-degree relatives at risk [19,20,27,30].

### 2.2. ATTR Polyneuropathy (ATTR-PN)

ATTR-PN results from the deposition of ATTR fibrils in the endoneurium of peripheral nerves, leading to progressive axonal and length-dependent neuropathy, sensory and motor deficits, and autonomic dysfunction (Table 2), which affects the patient’s quality of life [7,32,33]. The type and severity of neurological symptoms can vary greatly depending on the specific TTR variant, underscoring the importance of genetic testing for accurate diagnosis and patient-specific management [6,17,18,32]. Neurologic dysfunction is most prominent for the p.Val50Met variant, the most common TTR mutation worldwide [5,12].

#### 2.2.1. Symptoms and Neurological Red Flags

The ATTR-PN phenotype is highly variable and may include symptoms of peripheral neuropathy, including paresthesia and neuropathic pain in hands and feet; orthopedic and musculoskeletal manifestations such as bilateral carpal tunnel syndrome (CTS) and spinal stenosis; and symptoms of autonomic neuropathy, such as gastroparesis, diarrhea, constipation, bladder dysfunction, erectile dysfunction, and orthostatic hypotension (Table 2). These relatively non-specific neurological symptoms can often be overlooked or misattributed to other causes, such as diabetic or alcoholic polyneuropathy.

#### 2.2.2. Diagnostic Algorithm

If ATTR-PN is suspected on the basis of clinical symptoms and red flags, family history or endemic region (e.g., Portugal, Japan, Sweden or Brazil), polyneuropathy should be confirmed by a thorough neurological examination, which includes for example electrophysiological screening (Electroneuromyography—ENMG), test for sensory and/or motor involvement as well as reflex losses and dysautonomia, assessment of neuropathy impairment score (NIS) [12,17,18,32]. Since ATTRv-PN is treatable, Carrol et al. (2022) suggested a low threshold for early genetic testing [18]. With positive genetic testing and positive family history, ATTR-PN can be diagnosed without the need for biopsy and confirmation by IHC/MS, if the following criteria are met: monoclonal gammopathy can be excluded by hematological testing, scintigraphy shows Grade 2–3 cardiac uptake and findings in ECHO and CMR are consistent with ATTR-CM (Figure 2) [18].

Following Coutinho et al., the progression of ATTR-PN is staged according to the degree of ambulatory impairment: from stage 1 (unassisted walking) to stage 2 (walking with assistance) to stage 3 (wheelchair- and/or bed-bound) [34]. Please note that, according to the FDA and EMA, current therapies for ATTRv-PN are indicated for patients with Coutinho stage 1–2 neuropathy (see Section 4.1 for more details).

### 2.3. Mixed Phenotypes

Although ATTR patients have historically been classified according to their primary symptoms as either ATTR-PN or ATTR-CM, many present with both cardiac and neurological manifestations [5]. Of note, the proportion of symptomatic patients with mixed phenotype in the THAOS registry increased from approximately 17% (2022) to 25% (2023), ranging from 19% in North America to 41% in Asia (without Japan) [5,21]. This increase is mainly due to changes in the phenotype definitions that primarily affected the ATTRwt subgroup, leading to fewer patients classified as predominantly ATTRwt-CM. According to the most recent analysis in 2024, approximately one-third of symptomatic patients (33.5%) are classified as having a mixed phenotype, which is more common in ATTRv (75.7%) compared to ATTRwt amyloidosis (24.3%) [21]. Mixed phenotypes may generally be under-represented due to potentially less comprehensive neurological assessment in cardiac centers and vice versa (known as “referral bias”) [5]. Cardiologists may miss subtle neuropathic symptoms (Table 2), while neurologists may miss cardiac manifestations, highlighting the need for a multidisciplinary approach (Table 1). It is important to ensure that both cardiac and neurological investigations are carried out in suspected cases of ATTR.

It is important to note that, aside from tafamidis, most disease-modifying therapies have been approved specifically for either ATTR-PN (patisiran, inotersen, eplontersen vutrisiran) or ATTR-CM (acoramidis, vutrisiran). This leads to uncertainty about their efficacy in patients with a mixed phenotype. See Section 3 for more details and Section 6.2 for further discussions of treatment options for these patients.

## 3. Specific Therapies—Approved for ATTR Amyloidosis

In addition to symptomatic therapies—to manage disease-related symptoms such as heart failure, neuropathic pain, and autonomic dysfunction—there are a growing number of disease-modifying therapy options for ATTR-CM and ATTR-PN [35]. The specific therapies currently approved by regulatory authorities in the United States (US) and the European Union (EU) fall into two categories, based on their mode of action (Figure 1): Small molecule drugs (“TTR stabilizers”) bind to the TTR tetramer, thereby preventing tetramer dissociation, subsequent monomer misfolding, and amyloid fibril deposition [36]. Gene silencers are based on either small interfering RNA (siRNA) or antisense oligonucleotides (ASO) that specifically target TTR mRNA for degradation, effectively reducing the production of mutant and wild-type TTR protein [37,38]. See Section 5 for future therapeutic options in clinical development.

### 3.1. TTR Tetramer Stabilization

**Figure 3:**
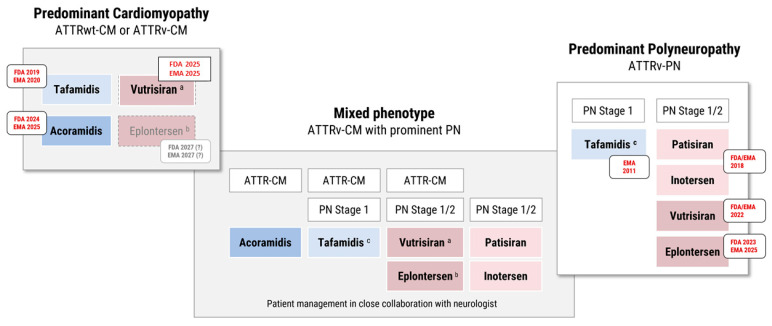
Approved and emerging therapeutic options for transthyretin (ATTR) amyloidosis with predominant cardiomyopathy (ATTR-CM), polyneuropathy (ATTR-PN), or mixed phenotype.Patients with ATTRv-CM and concurrent polyneuropathy (PN stage 1 or 2) should be managed in close collaboration with neurologists. Treatment selection should be guided by the extent of cardiac and neurological involvement and according to approved indications by local regulatory authorities. For predominant ATTR-CM (ATTRwt-CM or ATTRv-CM): • Tafamidis is approved by both FDA and EMA. • Acoramidis is approved for ATTR-CM in both the US and EU. • Vutrisiran is approved for ATTR-CM in both the US and EU. • Eplontersen is currently under evaluation for ATTR-CM in an ongoing phase III clinical trial (“CARDIO-TTRansform”). For predominant ATTRv-PN (stage 1 or 2): • Tafamidis is approved in the EU for stage 1 ATTRv-PN. • Patisiran, Inotersen, Eplontersen, Vutrisiran are approved in both the US and EU for ATTRv-PN (stage 1/2).

As mentioned, TTR stabilizers prevent the dissociation of the TTR tetramer into monomers by—as the name says—stabilizing the protein structure through interaction. Diflunisal is a nonsteroidal anti-inflammatory drug (NSAID) and non-specific TTR stabilizer in vitro that has been used off-label to treat ATTRv-PN [39,40]. However, the lack of approval and the increased risk of gastrointestinal, renal, and cardiac toxicity associated with the long-term use of NSAIDs make it unfavorable for ATTRv or ATTRwt patients with cardiac involvement [41,42]. Meanwhile, more advanced and selective TTR stabilizers have emerged. Tafamidis, a diflunisal derivative, binds to the thyroxine-binding sites of the TTR tetramer and prevents its dissociation through non-covalent interactions. This mechanism preserves the native tetrameric structure and inhibits amyloid fibril formation. Acoramidis represents a newer generation of stabilizers that also target the thyroxine-binding sites, but with a distinct molecular design aimed at mimicking the protective T119M variant. This allows for stronger binding affinity and enhanced pharmacological stabilization of TTR (Figure 3) [36].

#### 3.1.1. Tafamidis

Tafamidis is a small-molecule drug that specifically binds to the T4-binding site of TTR [43]. In 2019, tafamidis was approved in the US for the treatment of ATTR-CM. In the pivotal double-blind, placebo-controlled phase III trial ATTR-ACT (NCT01994889) including 441 patients with ATTR-CM (76% ATTRwt), tafamidis meglumine (20 mg) demonstrated a win ratio of 1.70 (95% CI: 1.26–2.29); with a 30% reduction in all-cause mortality and a 32% lower rate of cardiovascular (CV)-related hospitalization after 30 months compared to placebo [44]. Because these benefits were restricted to early-stage patients with New York Heart Association (NYHA) class ≤ II, the EMA first refrained from approving tafamidis for a broad ATTR-CM population [8,44]. In 2020, the European Commission granted marketing authorization for tafamidis (61 mg) in ATTR-CM [45].

Already in 2011, tafamidis meglumine (20 mg) gained approval in the European Union for stage 1 ATTR-PN based on its efficacy in patients with early neuropathy, where treatment has the greatest potential to preserve neurological function [45]. The EMA approval was based on the randomized, double-blinded phase III study Fx-005 (NCT00409175), where tafamidis was able to reduce disease progression in 60% of cases compared to 38% in the placebo group [43]. In contrast, tafamidis is not FDA-approved for ATTR-PN (Figure 3).

#### 3.1.2. Acoramidis

Acoramidis (formerly AG10) is an orally administered small-molecule drug specifically designed to mimic the protective T119M TTR variant—a naturally occurring mutation that enhances tetramer stability and reduces the risk of amyloidosis even in the presence of pathogenic variants. By reinforcing intermonomeric hydrogen bonding, including the formation of a stabilizing salt bridge with Ser117 [46], acoramidis achieves higher binding affinity and superior thermodynamic stabilization compared to tafamidis. In vitro studies further support these findings, demonstrating improved selectivity and a more potent stabilizing effect than both tafamidis and diflunisal [47]. By ensuring maximal and sustained TTR stabilization, these properties may offer a therapeutic advantage in preventing disease progression

In November 2024, the FDA approved acoramidis for ATTRwt and ATTRv patients with ATTR-CM [48]. In January 2025, acoramidis was approved in the EU for the same indication [49]. FDA and EMA approval are based on the randomized, double-blind, placebo-controlled phase III study ATTRibute-CM (NCT03860935), which demonstrated a significant benefit over placebo, with a win ratio of 1.8 (95% CI: 1.4–2.2), including a reduction in all-cause mortality and CV-related hospitalization [50] (Figure 3). In this study, 632 patients with ATTR-CM were randomly assigned to receive acoramidis (800 mg twice daily) or placebo for 30 months [50]. Long-term data up to 42 months showed that continued treatment with acoramidis resulted in a 43% reduction in all-cause mortality or first CV-related hospitalization compared to those who switched from placebo [51]. A randomized, double-blind, placebo-controlled phase III study (ACT-EARLY, NCT06563895) is going to evaluate the efficacy of acoramidis in preventing or delaying symptom onset of ATTR-PN or ATTR-CM in asymptomatic ATTRv patients.

### 3.2. TTR Gene Silencing

Gene silencers inhibit the expression of the TTR genes at the post-transcriptional level, strongly reducing the production of wild-type and variant TTR protein [37,38]. Small interfering RNAs (siRNAs) are double-stranded RNA molecules that bind to complementary mRNA sequences, leading to their degradation by the RNA-induced silencing complex (RISC) [37,38]. Antisense oligonucleotides (ASOs) are short, single-stranded RNA sequences that trigger mRNA degradation via RNase H [37,38]. Recently approved gene silencers for ATTR amyloidosis include the siRNA vutrisiran and the next-generation ASO eplontersen, which allow liver-specific delivery [38,52]. Clinical development of the investigational siRNA drug revusiran was discontinued in 2016 due to safety concerns during the phase III study (ENDEAVOUR; NCT02319005) [53,54].

Historically, liver transplantation was an early, albeit rather radical and invasive, form of “TTR gene silencing”, as hepatocytes produce approximately 95% of the circulating variant TTR protein [4,7]. Between the 1990s and the early 2000s, it was the first and primary treatment option for ATTRv amyloidosis with polyneuropathy. However, with the emergence of specific therapies, the importance and frequency of liver transplantation declined rapidly [32].

#### 3.2.1. Eplontersen (GalNAc-Conjugated ASO)

Eplontersen is a subcutaneous, ligand-conjugated successor of the first-generation ASO inotersen, which was approved for ATTRv-PN in 2018 by the FDA and EMA [52,55,56,57]. Conjugation with three N-acetylgalactosamine (GalNAc) residues enables liver-specific uptake of eplontersen by binding to the trivalent asialoglycoprotein receptor (ASGPR), which is predominantly expressed on hepatocytes [52,58,59]. In preclinical models, GalNAc-ASO conjugates demonstrated a 10-fold improvement in hepatic delivery compared to non-conjugated ASOs, resulting in a substantial dose reduction [58].

Since 2023, eplontersen has been approved in the United States for patients with ATTRv-PN stage 1/2, based on the positive results of the global, open-label, randomized phase III trial NEURO-TTRansform (NCT04136184) [60,61]. At week 65/66, ATTRv-PN patients treated once-monthly with eplontersen demonstrated a 70% reduction in serum TTR compared to placebo and a significant slowing of neuropathy progression, with −24.8 points difference to placebo in mNIS+7 [60]. Furthermore, quality of life significantly improved, as assessed by the Norfolk Quality of Life-Diabetic Neuropathy (Norfolk QoL-DN) questionnaire, with a difference of −19.7 points compared to placebo [60]. Eplontersen is self-administered once-monthly by subcutaneous injection via an autoinjector [62]. In March 2025, the European Commission approved eplontersen for the treatment of adults with stage 1 or stage 2 ATTRv-PN in the EU (Figure 3) [63].

The efficacy of eplontersen in patients with ATTR-CM (NYHA class I-III) is currently investigated in a global, double-blind, randomized, placebo-controlled phase III trial (CARDIO-TTRansform; NCT04136171) [24]. Patients receive either eplontersen (s.c. injection once every 4 weeks) or placebo, and efficacy is assessed using a primary composite endpoint of CV mortality and recurrent CV events at week 140. Secondary endpoints include all-cause mortality and change in 6MWT and KCCQ. CARDIO-TTRansform is the largest clinical trial in ATTR-CM to date, with an estimated 1438 patients enrolled [24]. Trial results are expected in mid-2026. In February 2024, eplontersen received FDA Fast Track designation for accelerated review for the indication ATTR-CM [64].

#### 3.2.2. Vutrisiran (GalNAc-Conjugated siRNA)

Vutrisiran is the subcutaneously administered successor of patisiran, the first siRNA drug gaining approval for ATTRv-PN in 2018 [65,66]. Like the ASO eplontersen, the siRNA vutrisiran is GalNAc-conjugated and designed for subcutaneous administration [67]. Vutrisiran has been evaluated in the global, open-label phase III study HELIOS-A (NCT03759379) [68]. The administration of vutrisiran (s.c. injection of 25 mg every 12 weeks) led to a slower decline in the neurological function and quality of life at Month 9 compared to placebo, as assessed by the modified Neuropathy Impairment Score+7 (mNIS+7) and the Norfolk Quality of Life-Diabetic Neuropathy (Norfolk QoL-DN) questionnaire [68]. In 2022, vutrisiran was approved by the FDA and EMA for patients with stage 1 and stage 2 ATTRv-PN (Figure 3) [69,70].

In the randomized, double-blind phase III trial HELIOS-B (NCT04153149), vutrisiran demonstrated efficacy in 655 patients with ATTR-CM, leading to a 28% lower risk of all-cause mortality and recurrent CV events at 36 months than placebo [71]. Secondary outcome measures significantly improved, including NYHA class, six-minute walk test (6MWT), and Quality of Life (QoL), as assessed by the Kansas City Cardiomyopathy Questionnaire Clinical Summary Score (KCCQ-CSS) [71]. Vutrisiran is approved by the FDA and EMA for the treatment of ATTR-CM (Figure 3) [69,70].

## 4. Treatment Strategies Based on ATTR Phenotype

A multidisciplinary approach is essential, including symptomatic therapy to relieve symptoms and improve quality of life and disease-modifying therapy to slow disease progression [9,72]. In ATTR-CM, symptomatic management focuses on HF symptoms, arrhythmias, and hypotension, while taking into account ATTR-specific pathophysiology such as restrictive cardiomyopathy and intolerance to most of the standard HF treatments (except, for example, SGLT2 inhibitors) [27,30,73,74,75,76]. In general, the symptomatic management of ATTR-CM follows the CHAD-STOP concept: Indicated are conduction and rhythm disorders prevention, high heart rate maintenance, anticoagulation, and diuretics (CHAD), whereas the following medications should be used with caution (STOP): beta-receptor- and calcium-channel blockers, digoxin, and renin-angiotensin-aldosterone [77,78]. Symptomatic management of ATTR-PN is primarily aimed at relieving neuropathic pain, improving mobility, and treating autonomic dysfunction. The result is a substantial improvement in the patient’s quality of life. For further details on the management of patients with ATTRv-PN, please refer to current guidelines and expert recommendations [17,18,32].

The rapid pace of drug development makes it difficult for medical associations, guideline authors, and physicians to keep up with the latest developments and approvals. The lack of head-to-head trials and the limited FDA/EMA approval for patients with either predominant cardiomyopathic (ATTR-CM) or predominant neuropathic (ATTR-PN) manifestations in the early stages complicates the decision for comprehensive therapy measures, especially for patients with advanced-stage disease and mixed phenotype.

### 4.1. Patients with ATTR-PN

To date, four gene silencers are approved by the FDA and EMA for the treatment of patients with stage 1 or stage 2 ATTRv-PN: patisiran (I.V. siRNA), vutrisiran (s.c. GalNAc-siRNA), inotersen (s.c. ASO), and eplontersen (s.c. self-administered GalNAc-ASO). In the EU, the TTR stabilizer tafamidis meglumine (20 mg) has approval for ATTRv-PN stage 1. Please note that tafamidis is FDA approved for ATTR-CM but not for ATTR-PN.

Depending on the stage of neuropathy and local regulatory approval, ATTR amyloidosis patients with hereditary polyneuropathy (ATTRv-PN) can be treated with either tafamidis (stage 1) or gene silencers such as vutrisiran or eplontersen (stage 1/2; the predecessor drugs patisiran or inotersen are less frequently used in clinical routine). For patients with additional ATTR-CM, see Section 4.3.

### 4.2. Patients with ATTR-CM

According to the 2021 ESC Guideline and the 2023 ACC Expert Consensus Decision, ATTR-CM and NYHA class I-II can be treated with the TTR stabilizer tafamidis. In the AHA/ACC/HFSA guideline for the management of HF (2022), tafamidis is recommended for patients up to NYHA class III to reduce CV-related morbidity and mortality [74]. Recently, a second stabilizer, acoramidis, has been approved for these patients [48,49]. The selection between tafamidis and acoramidis for treating ATTR-CM should be informed by both clinical trial data and individual patient characteristics. Tafamidis has demonstrated a significant reduction in all-cause mortality and cardiovascular hospitalizations in the ATTR-ACT trial, with pronounced benefits in NYHA class I–II patients. Acoramidis, evaluated in the ATTRibute-CM trial, showed improvements in functional capacity and quality of life, with non-inferiority in mortality endpoints, although a clear survival benefit has yet to be established. Notably, acoramidis achieves near-complete (>90%) transthyretin (TTR) stabilization, as evidenced by rapid and sustained increases in serum TTR levels. In the ATTRibute-CM study, patients exhibited an average increase of 9.1 mg/dL in serum TTR within 28 days of initiating acoramidis, an effect maintained over the 30-month trial period. Further analysis indicated that each 5 mg/dL increase in serum TTR was associated with up to a 31.6% reduction in mortality risk over 30 months, suggesting that serum TTR levels could serve as a prognostic biomarker for treatment response. While no head-to-head comparisons exist between tafamidis and acoramidis, both agents effectively stabilize TTR. In clinical practice, treatment decisions will likely be influenced by factors such as drug availability, cost, renal function, dosing frequency, and patient preference. Tafamidis remains a well-established first-line therapy with robust long-term outcome data. However, acoramidis presents a promising alternative, particularly for patients who may benefit from its potent TTR stabilization and the potential for monitoring serum TTR levels as a marker of therapeutic efficacy. Ongoing and future real-world studies will be essential to further delineate the comparative effectiveness of these therapies.

Of note, vutrisiran (HELIOS-B; approved in 2025 by FDA and EMA for ATTR-CM) and eplontersen (CARDIO-TTRansform) have completed or ongoing phase III trials in ATTR-CM, which makes both an attractive alternative—not only in ATTRwt-CM, but also for mixed phenotype patients with additional PN (Section 4.3, (Figure 3). See Section 6.2 for further discussion.

### 4.3. Patients with Mixed Phenotype

Although ATTR amyloidosis is a highly heterogeneous disease, most TTR stabilizers and gene silencers are currently indicated for a predominant phenotype. This raises an important and urgent question: How to choose the best first-line therapy for patients with mixed phenotype, who make up one-third of symptomatic patients [21]?

Patients with ATTRv-CM and additional polyneuropathy can be treated with TTR stabilizers, as tafamidis and acoramidis are both FDA and EMA approved for ATTR-CM. However, it is important to remember that EMA approval of tafamidis is restricted to ATTR-PN stage 1, based on the results of the FX-005 trial, and that neither tafamidis nor acoramidis is FDA approved for isolated ATTR-PN. In addition, gene silencers may be considered for ATTR-CM patients with mixed phenotypes and pronounced neurological impairment, which requires close collaboration with neurologists.

## 5. Future Therapeutic Options

### 5.1. TTR Gene Editing

The CRISPR-Cas9 (clustered regularly interspaced short palindromic repeats and associated Cas9 endonuclease) gene-editing system has revolutionized genetic engineering by providing a precise and efficient tool for modifying DNA [38]. It has enabled groundbreaking advances in biomedical research and therapeutic development, offering potential once-in-a-lifetime treatments for life-threatening genetic disorders [37,38]. As of March 2025, there is only one CRISPR-Cas9-based therapy approved for the treatment of sickle cell disease [79]. Several other gene therapies are currently in phase III clinical trials for other fatal diseases, including ATTR amyloidosis [80].

#### Nexiguran Ziclumeran

Nexiguran ziclumeran (nex-z; previously NTLA-2001) was the first gene editing therapy based on CRISPR-Cas9 under clinical investigation in humans [37,81]. An open-label phase I trial (NCT04601051) investigated the safety and efficacy of a single I.V. infusion of nex-z in patients with ATTR-CM [81]. After 12 months, the serum TTR concentration was reduced by 90%, with stable levels of NT-proBNP and cardiac troponin T. 92% of patients showed improvement or no change in NYHA class. Nex-z was well-tolerated, with infusion-related reactions as the most common adverse events [81]. A multinational, double-blind, placebo-controlled phase III trial (MAGNITUDE; NCT06128629) started at the end of 2023 and is anticipated to enroll about 765 patients. Patients are randomly assigned to receive a single I.V. infusion of nex-z or placebo. The primary endpoint is a composite outcome of CV mortality and CV events; secondary outcome measures are the change in serum TTR and KCCQ-OS score at month 18. Completion of the phase III trial is expected in 2028.

### 5.2. Anti-ATTR Antibodies

The aforementioned therapies focus on preventing amyloid fibril formation by stabilizing the TTR tetramer or reducing TTR production. More recently, therapeutic strategies have emerged that specifically target pathological ATTR deposition [7,35]. The main goal of these “TTR depleters” is to reverse the course of the disease and prevent re-accumulation, especially in patients with advanced disease. One notable approach is the development of monoclonal antibodies (mAbs) designed to specifically bind to misfolded TTR and mark the amyloid fibrils for clearance by phagocytes. As a result, the amyloid fibrils in the heart and the peripheral nervous system are effectively removed [82,83]. Two anti-ATTR mAbs, Coramitug and ALXN2220, have entered clinical development (Figure 1).

#### 5.2.1. Coramitug

Coramitug (NNC6019-0001; formerly PRX004) is a humanized IgG mAb designed to deplete ATTR fibril deposits that is currently being investigated in a global phase II trial (NCT05442047) in patients with ATTR-CM (NYHA II-III) [84,85]. Patients receive either Coramitug (two different doses by I.V. infusion every 4 weeks) or placebo added to the standard of care. Primary endpoints are the changes in 6MWT and NT-proBNP until week 52. Secondary endpoints include time to all-cause mortality, number of CV events, and changes in cardiomyopathy score (KCCQ-CSS) and neuropathy impairment score (NIS). The phase II study is expected to be completed in mid-2025.

#### 5.2.2. ALXN2220

ALXN2220 (formerly NI006 and NI301A) is a recombinant human IgG mAb that selectively binds with high affinity to a linear epitope (WEPFA) accessible only on misfolded TTR and amyloid fibril aggregates of both ATTRv and ATTRwt [83,86]. Following a successful phase I study (NCT04360434) in patients with ATTR-CM, ALXN2220 is currently being evaluated in the global, randomized, double-blind, placebo-controlled phase III study (DepleTTR-CM; NCT06183931) [87]. The study will enroll an estimated 1000 patients with ATTR-CM and a history of HF (NYHA II-IV) and is expected to be completed in 2028. Efficacy will be assessed by the occurrence of all-cause mortality and CV events up to month 48, with changes in KCCQ-OS and 6MWT as secondary endpoints.

## 6. How to Further Improve Diagnosis and Therapy?

### 6.1. Earlier Diagnosis and Better Access to Specialized Centers

ATTR amyloidosis is a rare disease with multisystemic manifestations. The combination of low disease awareness and heterogeneous symptoms (which are non-specific in isolation but can be specific for ATTR amyloidosis in combination) often leads to delayed or missed diagnosis. Hence, cardiologists should be encouraged to recognize neurological and other extracardiac red flags, and neurologists should consider cardiac involvement that could be associated with ATTR amyloidosis. Other experts, including gastroenterologists, surgeons, or orthopedic surgeons, should be involved in diagnostic steps and treatment decisions on an individual patient basis in order to provide the best possible and comprehensive care. Especially orthopedic surgeons should consider ATTR amyloidosis in patients undergoing surgery [9,19,72,88]. With the increasing number of recently developed and approved disease-modifying therapies capable of slowing disease progression, patients with suspected ATTR amyloidosis stand to benefit greatly from accurate, timely, and subtype-specific diagnosis.

Historically, the histological detection of amyloid deposits has been the gold standard for diagnosing ATTR amyloidosis. However, advances in diagnostic methods have made non-invasive approaches increasingly feasible and effective, and more suitable for patients who are not yet symptomatic or at an early disease stage. Among non-invasive methods, 99mTc-scintigraphy with tracers such as DPD, HMDP, or PYP combined with SPECT, as well as CMR imaging, offers the highest diagnostic accuracy after exclusion of monoclonal gammopathy. Screening for monoclonal gammopathy included the combination of serum electrophoresis, free light-chain assay, and immunofixation of serum and urine. However, CMR is especially expensive and less accessible in low-resource settings [89]. Hence, there is an urgent need for more broadly available alternatives, e.g., sensitive and ATTR-specific blood tests and biomarkers (“liquid biopsy”). These blood tests could be used to screen for ATTR-CM in high-risk populations, such as elderly patients with symmetric polyneuropathy, HFpEF, aortic stenosis, lumbar spinal stenosis, or bilateral carpal tunnel syndrome [11,31,90,91,92].

Artificial intelligence (AI) and machine learning are increasingly being integrated into the diagnosis of cardiac amyloidosis. Recent studies have demonstrated the potential of AI to enhance diagnostic accuracy and facilitate early detection [93,94,95]. Automated echocardiographic analysis tools, such as US2AI software (version 2), have emerged as powerful solutions for enhancing the efficiency and accuracy of echocardiogram interpretation [96]. Ongoing research and development in this field are expected to further integrate AI into clinical practice, improving the rate of early diagnosis and finally patient outcomes. AI-based tools not only support diagnosis and prognostication but may also contribute to earlier detection in asymptomatic individuals or those at elevated risk, based on subtle ECG or imaging changes. In general, there should be a low threshold for early genetic testing. Unfortunately, there are considerable regional differences in access to genetic testing and eligibility for reimbursement. Amyloidosis ‘centers of excellence’ can provide the clinical knowledge and capacity for genetic testing and other diagnostic methods [97]. Therefore, minimizing barriers to accessing specialized amyloidosis centers is essential to ensure timely diagnosis and optimal care for patients with suspected ATTR amyloidosis (https://www.amyloidosissupport.org/amyloidosis_centers.html, accessed on 30 June 2025).

### 6.2. Improving Strategies for Individualized Treatment Approaches

The regulatory requirement to conduct two separate clinical trials for the treatment of a single disease represents a significant cost factor and is relatively uncommon, particularly in the context of rare, multisystemic disorders such as Fabry or Gaucher disease. The indications for an approved therapy are typically based on the inclusion criteria of the clinical trials that supported its approval. However, trial design is heavily influenced by regulatory authorities, resulting in a unique regulatory framework for ATTR amyloidosis. As recently pointed out by Fine and Witteles, this paradigm results in a “limited and artificial approach to treatment decisions” [98]. In clinical practice, approximately one-third of symptomatic patients do not fit neatly into either of the two traditional phenotype categories—ATTR-CM or ATTR-PN. This underscores the need for a more flexible and personalized approach to treatment decisions that better reflects the diverse manifestations of the disease. Until recently, most specific therapies were only approved for early-stage ATTRv, with tafamidis being the only option for patients with ATTR-CM. However, the therapeutic landscape for ATTR-CM has expanded significantly: It now includes another approved TTR stabilizer (acoramidis) as well as two gene silencers with positive (vutrisiran; approved by FDA and EMA) or ongoing phase III trial (eplontersen) in patients with ATTR-CM.

The introduction of additional treatment options raises new questions: Which treatment should be the first choice for a given patient? How should we define and manage non-responders? What are useful and meaningful indicators of treatment response: change in serum TTR, alongside functional, imaging, and biomarker assessments (e.g., NT-proBNP, troponin, echocardiography)? Given the different modes of action, does it make sense to switch to a different drug class for patients who do not respond or experience significant side effects, or even to combine two different therapies? The considerable clinical heterogeneity highlights the need for individualized treatment. However, this also complicates the selection of the optimal therapy, as the study populations in different clinical trials are not directly comparable. A practical approach in clinical practice could be to compare the individual patient with study populations from existing trials based on factors such as disease stage, phenotype, genetic background, and comorbidities. While a greater similarity of an individual patient to the population enrolled in trial A versus trial B may provide some rationale for preferring one therapeutic option over another, it is unlikely to serve as a primary criterion for treatment selection in clinical practice. In the absence of direct comparative data, decisions will most likely be guided by a combination of factors, including drug pricing, renal function, dosing regimen, and individual patient characteristics.

Could it even be beneficial to combine two different therapeutic approaches for certain patients, for example, those with mixed phenotypes? The phase III HELIOS-B study of vutrisiran was not powered to demonstrate a statistically significant difference in patients receiving tafamidis at baseline, making it difficult to assess the additional benefit of vutrisiran in this setting. However, the ongoing CARDIO-TTRansform trial of eplontersen, which is enrolling >1400 ATTR-CM patients with or without current tafamidis treatment, may provide valuable insights into the efficacy of combination therapy compared to monotherapy. As noted above, the development of specific therapies requires extensive clinical research to demonstrate efficacy, which is associated with high healthcare costs and limited accessibility, particularly in low-resource settings. As a result, combination therapy may be limited in regions where patients rely on public reimbursement for access to treatment.

The off-label use of non-specific TTR stabilizers, such as diflunisal or tolcapone, should only be considered when approved, more targeted treatment options are unavailable. Nonsteroidal anti-inflammatory drugs, including diflunisal, should be used with great caution in patients with ATTR-CM, especially those with advanced HF and/or significant renal impairment, as they may exacerbate these conditions.

Therapy adherence is another critical factor to consider. Oral therapies, such as tafamidis (once daily) and acoramidis (twice daily), are well-suited for patients who prioritize convenience and can commit to a regular daily regimen. In contrast, infusion therapies like patisiran or vutrisiran require clinic visits every three months, which may pose challenges for patients with busy lifestyles or limited access to healthcare facilities. Subcutaneous options provide greater flexibility with less frequent administration. As the successor to inotersen, eplontersen is the first ATTR-specific therapy that offers self-administration once monthly via an auto-injector, potentially improving adherence and accessibility for patients.

### 6.3. Future Options for Advanced and Asymptomatic Patients

Existing therapeutic options are mainly aimed at slowing disease progression, with efficacy best documented in early to mid-stage disease, i.e., ATTR-CM with NYHA class I-II or ATTRv-PN stage 1–2. However, these therapies do not cure, halt, or reverse the disease. Consequently, treatment options remain limited for advanced ATTR-CM and ATTR-PN. Promising developments include antibody-based “ATTR depleters”, such as coramitug (currently in phase II trials) and ALXN2220 (in phase III trials). These therapies have the potential to clear existing amyloid deposits, particularly in older, late-stage ATTR-CM patients (NYHA III-IV). In addition, future clinical trials, such as the ACT-EARLY trial of acoramidis, may determine whether certain therapies can prevent or delay disease onset in asymptomatic or early-symptomatic patients, offering hope for improved outcomes at earlier stages of the disease.

In conclusion, Amyloidosis is no longer untreatable. Improved awareness, earlier diagnosis, and disease-modifying therapies, including gene silencing and TTR stabilization, have changed the trajectory of this condition. However, access to modern diagnostics and timely recognition remain key challenges.

## Figures and Tables

**Table 1:** Red Flags for Suspected ATTR-CM.

	Cardiac Symptoms and Red Flags of ATTR-CM
Clinical	Symptoms of heart failure—often with preserved ejection fraction (HFpEF), but occasionally with reduced EF—include dyspnea, fatigue, peripheral edema, palpitations, exercise intolerance, angina pectoris
ECHO	LV thickening (≥12 mm; without arterial hypertension), granular sparkling, abnormal longitudinal strain with apical sparing, aortic stenosis
CMR	Diffuse late gadolinium enhancement (LGE), highly increased native T1
ECG	Pseudo-infarct pattern, prolonged QTc, low QRS voltage (despite LV thickening), conduction disease, atrial fibrillation
Biomarkers	Elevated BNP/NT-proBNP, persistently increased cardiac troponins (cTnT, cTnI)

**Table 2:** Additional clinical indicators that should raise suspicion of ATTR.

	Extracardiac Manifestations and Neurological Red Flags
Orthopedic/ Musculoskeletal	Bilateral carpal tunnel syndrome (CTS), spinal stenosis (lumbar or cervical), rupture of the distal biceps tendon
Peripheral Neuropathy	Paresthesia and neuropathic pain in feet and hands (e.g., loss of sensation, numbness, tingling, burning sensation)
Autonomic Dysfunction	Chronic diarrhea, weight loss, constipation, early satiety, orthostatic hypotension, erectile dysfunction, urinary retention or incontinence, sweating abnormalities
